# The relationship between polymorphisms of microRNA and preeclampsia: A protocol for meta-analysis and bioinformatics prediction

**DOI:** 10.1097/MD.0000000000025477

**Published:** 2021-04-09

**Authors:** Tao Li, Yihong Chen, Yi Lai, Guoqian He, Guolin He

**Affiliations:** a Key Laboratory of Birth Defects and Related Diseases of Women and Children, Ministry of Education; b Department of Obstetrics and Gynecology, West China Second University Hospital, Sichuan University, Chengdu, Sichuan, China.

**Keywords:** meta-analysis, microRNA, preeclampsia, protocol

## Abstract

**Background:**

Preeclampsia has genetic correlation. Many studies have shown that microRNA (miRNA) polymorphism is highly associated with preeclampsia, but the results are inconsistent. The purpose of this study is to systematically evaluate the relationship between miRNA polymorphism and preeclampsia.

**Methods:**

In this study, the search time is set from the establishment of the database on January 2021. The search database include China National Knowledge Infrastructure (CNKI), Wanfang, VIP and China Biology Medicine disc (CBM), PubMed, EMBASE, and Web of Science, and the Cochrane Library. The subjects are case-control studies on the relationship between miRNA polymorphism and preeclampsia. The language is limited to English and Chinese. The data of the included study are extracted and the literature quality is evaluated by 2 researchers independently. The data are statistically analyzed through Stata 16.0 software. We also predicted the miRNA secondary structure and the binding sites of miRNA interaction with its target genes

**Results:**

This review will be disseminated in print by peer-review.

**Conclusion:**

This study will provide evidence-based medicine to elucidate the genetic tendency of preeclampsia.

**Ethics and dissemination:**

Private information from individuals will not be published. This systematic review also does not involve endangering participant rights. Ethical approval will not be required. The results may be published in a peer-reviewed journal or disseminated at relevant conferences.

**OSF Registration number:**

DOI 10.17605/OSF.IO/MJY2X.

## 1 Introduction

Preeclampsia is a multisystem disease that occurs in 2% to 10% of pregnant women and is the leading cause of maternal and perinatal morbidity and mortality.^[1,2]^ Preeclampsia usually occurs after 20 weeks of pregnancy, accompanied with hypertension and proteinuria, which may be caused by insufficient blood perfusion and ischemia due to placental defects.^[3,4]^ In addition, perhaps, preeclampsia is the main cause of maternal and infant morbidity, preterm delivery, and fetal growth restriction.^[5–7]^ Most studies have revealed that insufficient trophoblast invasion into the uterine artery results in a decrease in uterine-placental perfusion. Meanwhile, placental ischemia and changes in maternal immune response may play important roles in the development of preeclampsia. Although extensive studies have been carried out, accurate diagnostic markers for preeclampsia^[8,9]^ is still scarce.

MicroRNA (miRNA) is a non-coding single-stranded RNA and participates in the regulation of apoptosis, proliferation, differentiation, ontogeny, and other life activities.^[10]^ By regulating many target genes, miRNA participates in the process of immune response and plays a very important role in the occurrence and development of autoimmune diseases.^[11,12]^ miRNA not only plays a significant role in the normal growth and differentiation of body cells, but also participates in pathological processes such as inflammatory reaction and carcinogenesis. At the same time, miRNA plays an important role in the occurrence and development of malignant tumors such as liver cancer, breast cancer, and rectal cancer.^[13,14]^ Trophoblast invasion is very similar to tumor cell infiltration during pregnancy. Recent studies have proved that miRNA is also important in the occurrence and development of preeclampsia.^[15]^ miRNA stably exists in blood and can be detected in patients’ serum to observe its expression.^[16]^

In different stages of pregnancy, a large number of miRNAs were found, and expression profiles were different between preeclampsia and normal placenta.^[17]^ Therefore, changing the level of miRNA may lead to the abnormal expression of target genes, abnormal placental function, and subsequent complications such as preeclampsia.^[17,18]^ By targeting genes in inflammatory response, miRNA-499 has potential inflammatory inhibitory effects and may play a regulatory role under the hypoxic-ischemic condition.^[19,20]^ The relationship between miRNA gene variation and many diseases has been confirmed, including the effects on pregnancy complications such as preeclampsia.^[21–23]^ Mohseni et al^[24]^ displayed that the expression of miRNA is higher in patients with preeclampsia and cardiac remodeling, suggesting that miRNA may play a role in cardiomyocyte biology. Alipour et al^[25]^ reported that miRNA is associated with recurrent pregnancy loss, some of which may be closely related to preeclampsia. Jeon et al^[26,27]^ found that miRNA polymorphism is associated with idiopathic, recurrent, and spontaneous abortion and fetus.

The abnormal expression of miRNA plays an important role in the occurrence and development of preeclampsia, which clearly indicates that miRNA polymorphism can be applied as a biomarker to evaluate the risk of preeclampsia. Many studies have explored the relationship between miRNA polymorphism and the risk of preeclampsia. However, the results of these studies are not consistent.^[28]^ Therefore, we conducted a meta-analysis to examine the accurate correlation between miRNA polymorphism and susceptibility to preeclampsia.

## 2 Methods

### 2.1 Protocol register

This protocol of systematic review and meta-analysis have been drafted under the guidance of the preferred reporting items for systematic reviews and meta-analyses protocols (PRISMA-P).^[29]^ Moreover, it has been registered on open science framework (OSF) on January 2021 (Registration number: DOI 10.17605/OSF.IO/MJY2X).

### 2.2 Ethics

Since the program does not include the recruitment of patients and the collection of personal information, it does not require the approval of the Ethics Committee.

### 2.3 Eligibility criteria

(1) The subjects were clinically diagnosed as preeclampsia with systolic blood pressure ≥140 mmHg and/or diastolic blood pressure ≥90 mmHg, and proteinuria ≥0.3 g for 24 hours or random urine protein ≥(+) after 20 weeks of pregnancy;
(2) The exposure factor was miRNA mutation;
(3) Outcome measures were the risk of preeclampsia;
(4) The control group involved pregnant women without hypertension;
(5) The study was a case-control study;
(6) Languages were limited to Chinese and English;
(7) Allele or genotype distribution frequency data were available;
(8) The distribution frequency of genotype conformed to Hardy-Weinberg's law.

### 2.4 Exclusion criteria

(1) Repeated published researches;
(2) In articles, the published literatures were abstracts or reviews, the data of the article were incomplete or incorrect, and the complete data cannot be obtained after contacting the author(s);
(3) The study failed to provide detailed data on the frequency of genotypes;
(4) Literatures had no relevant outcome indicators;
(5) The objects of studying were not from human beings.

### 2.5 Retrieval strategy

The search database include China National Knowledge Infrastructure (CNKI), Wanfang, VIP and China Biology Medicine disc, PubMed, EMBASE, and Web of Science and the Cochrane Library. All the literatures about the relationship between miRNA polymorphism and preeclampsia will be collected from the establishment of the database on January 2021. Taking PubMed as an example, the retrieval strategy is exhibited in Table 1.

**Table 1:** Search strategy in PubMed database.

Number	Search terms
#1	Pre-Eclampsia [MeSH]
#2	Toxemias, Pregnancy [Title/Abstract]
#3	EPH Complex [Title/Abstract]
#4	EPH Gestosis [Title/Abstract]
#5	EPH Toxemias [Title/Abstract]
#6	Edema-Proteinuria-Hypertension Gestosis [Title/Abstract]
#7	Gestosis, EPH [Title/Abstract]
#8	Hypertension-Edema-Proteinuria Gestosis [Title/Abstract]
#9	Preeclampsia [Title/Abstract]
#10	Preeclampsia/Eclampsia 1 [Title/Abstract]
#11	Pregnancy Toxemias [Title/Abstract]
#12	Proteinuria-Edema-Hypertension Gestosis [Title/Abstract]
#13	Toxemia Of Pregnancy [Title/Abstract]
#14	EPH Toxemia [Title/Abstract]
#15	Edema Proteinuria Hypertension Gestosis [Title/Abstract]
#16	Gestosis, Edema-Proteinuria-Hypertension [Title/Abstract]
#17	Gestosis, Hypertension-Edema-Proteinuria [Title/Abstract]
#18	Gestosis, Proteinuria-Edema-Hypertension [Title/Abstract]
#19	Hypertension Edema Proteinuria Gestosis [Title/Abstract]
#20	Pre Eclampsia [Title/Abstract]
#21	Pregnancy Toxemia [Title/Abstract]
#22	Proteinuria Edema Hypertension Gestosis [Title/Abstract]
#23	Toxemia, EPH [Title/Abstract]
#24	Toxemia, Pregnancy [Title/Abstract]
#25	Toxemias, EPH [Title/Abstract]
#26	or/1–26
#27	miRNA [Title/Abstract]
#28	microRNA [Title/Abstract]
#29	or/27–28
#30	polymorph∗[Title/Abstract]
#31	susceptibility[Title/Abstract]
#32	or/30–31
#33	#26 and #29 and #32

### 2.6 Data screening and extraction

**Figure 1:**
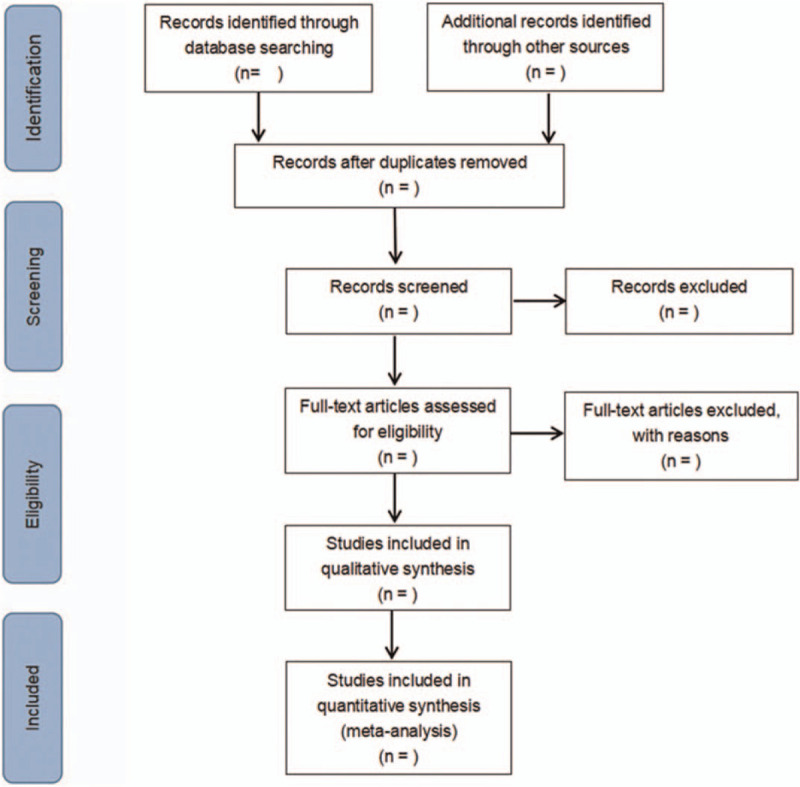
Flow diagram.

Two researchers independently complete the literature screening, exclude the studies that obviously do not meet the inclusion criteria, and further read the abstracts and the full texts to determine whether they meet the inclusion criteria. The data included in the literature will be extracted and cross-checked. Disagreement should be solved by consulting a third researcher, thus reaching a consensus. The extracted data include: the first author, the number of years of publication, the country of publication, the race of the study population, the basic characteristics of the study population (including age, sex, disease, etc), the distribution of each phenotype of genes (whether obeying the law of Hardy-Weinberg equilibrium), and so on. The literature screening process is illustrated in Fig. 1.

### 2.7 Literature quality assessment

The case-control study will be evaluated by Newcastle-Ottawa Scale (NOS), including 3 columns and 8 items with a total of 9 points, and the evaluation criteria ≥6 is regarded as high quality.

### 2.8 Statistical analysis

#### 2.8.1 Data analysis and processing

Before evaluating the correlation between miRNA polymorphism and preeclampsia, chi-squared will be adopted to test whether the genotype distribution in the control group is in accordance with Hardy-Weinberg genetic equilibrium (*P *≥ .05). Meta-analysis is carried out by using STATA.16 software (StataCorp LLC, college station, TX). The binary classification variables will be expressed as odds ratio (OR) and 95% confidence interval (CI). Heterogeneity is analyzed by conducting *Q* test and *I*^2^, and the heterogeneity is evaluated based on the *I*^2^ value. If *P* > .1 and *I*^2^ < 50%, the heterogeneity among the included studies is small, so the fixed effect model is applied for analysis. If *P* < .1 and *I*^*2*^ ≥ 50%, the heterogeneity among the included studies is obvious, and the sources of heterogeneity are analyzed. Random effect model will be utilized for analysis. RNAfold web server (http://rna.tbi.univie.ac.at/cgi-bin/RNAWebSuite/RNAfold.cgi/) was used to perform in silico analyses for the prediction of each miRNA secondary structure, harboring selected single nucleotide polymorphisms. miRNA binding sites were predicted by miRanda (http://www.mirbase.org/).

#### 2.8.2 Dealing with missing data

If the data of the required study are incomplete or not reported in the study, the researcher will contact the first author or other authors of the study by phone or email. If the required data are not available, we will perform descriptive analysis instead of meta-analysis and exclude these studies if necessary.

#### 2.8.3 Subgroup analysis

In order to deal with the heterogeneity among different studies, subgroup analyses will be conducted on the basis of ethnicity.

#### 2.8.4 Sensitivity analysis

In order to test the stability of the meta-analysis results, we will adopt the one-by-one exclusion method to analyze the sensitivity of the results.

#### 2.8.5 Assessment of reporting biases

A funnel chart will be used to qualitatively identify publication bias, and Egger and Begg tests will be applied to quantitatively evaluate publication bias. If the funnel diagram is asymmetrical, with *P* < .05, it is considered to have obvious publication bias.

## 3 Discussion

Preeclampsia is the occurrence of hypertension and proteinuria after 20 weeks of pregnancy with normal blood pressure before pregnancy. Preeclampsia is one of the hypertensive disorders of pregnancy and an idiopathic disease of pregnancy. Meanwhile, it is closely related to the maternal and fetal outcome.^[30]^ The incidence of preeclampsia is 2.5%, accounting for 6% of perinatal maternal death.^[31,32]^ The exact pathogenesis of preeclampsia is still unclear. Many scholars believe that preeclampsia has a familial genetic tendency and is a polygenic genetic disease. miRNA is a single-stranded RNA that does not encode a protein. At present, many studies have revealed that a close relationship between miRNA and the occurrence and development of preeclampsia can be found, while the relationship between miRNA polymorphism and the occurrence of preeclampsia is controversial. This study comprehensively searched the case-control study on the relationship between miRNA polymorphism and the risk of preeclampsia, and made a quantitative comprehensive evaluation by carrying out meta-analysis to provide evidence-based reference for the study on the etiology of preeclampsia.

This study also has some limitations. First of all, ethnic groups of meta-analysis are wide, while researches on single race are few. Population confounding factors may affect the credibility of the results in this study. Second, the method adopted in this study is meta-analysis that belongs to the secondary analysis. Therefore, the quality of this study mainly depends on the overall quality of the included studies, which is an inevitable problem of the secondary analysis. Third, due to the limitation of the number of studies included, there may be some publication bias in this meta-analysis.

To sum up, this meat-analysis will provide the correlation between miRNA polymorphism and the risk of preeclampsia. In view of the limitation in terms of the number of included studies, this conclusion needs to be verified through further large sample and high-quality studies, so as to clarify the relationship between gene polymorphism and preeclampsia.

## Author contributions

**Conceptualization:** Tao Li, Guolin He.

**Data curation:** Tao Li, Yihong Chen.

**Funding acquisition:** Guolin He.

**Investigation:** Guolin He.

**Methodology:** Yihong Chen.

**Project administration:** Guolin He.

**Resources:** Yihong Chen.

**Software:** Yihong Chen, Yi Lai.

**Supervision:** Yi Lai, Guoqian He.

**Validation:** Yi Lai, Guoqian He.

**Visualization:** Yi Lai.

**Writing – original draft:** Tao Li, Guolin He.

**Writing – review & editing:** Tao Li, Guolin He.
